# A cell membrane-targeting AIE photosensitizer as a necroptosis inducer for boosting cancer theranostics†

**DOI:** 10.1039/d2sc01260j

**Published:** 2022-04-19

**Authors:** Niu Niu, Ying Yu, Zhijun Zhang, Miaomiao Kang, Lei Wang, Zheng Zhao, Dong Wang, Ben Zhong Tang

**Affiliations:** Center for AIE Research, Shenzhen Key Laboratory of Polymer Science and Technology, Guangdong Research Center for Interfacial Engineering of Functional Materials, College of Material Science and Engineering, Shenzhen University Shenzhen 518060 China wangd@szu.edu.cn; College of Physics and Optoelectronic Engineering, Shenzhen University Shenzhen 518060 China; School of Science and Engineering, Shenzhen Institute of Aggregate Science and Technology, The Chinese University of Hong Kong Shenzhen Guangdong 518172 China tangbenz@cuhk.edu.cn

## Abstract

The exploration of cellular organelle-specific anchoring photosensitizers with both prominent fluorescence imaging behavior and extraordinary reactive oxygen species (ROS) production capability is highly in demand but remains a severe challenge for effective cancer theranostics involving photodynamic therapy (PDT). In this contribution, we developed a cell membrane-targeting and NIR-emission photosensitizer having an aggregation-induced emission (AIE) tendency. The AIE photosensitizer, namely TBMPEI, is capable of lighting up and ablating cancer cells by means of a necroptosis procedure enabling cell membrane rupture and DNA degradation upon light irradiation, endowing TBMPEI with impressive performance for both *in vitro* and *in vivo* fluorescence imaging-guided PDT.

## Abstract 2

A membrane-targeting photosensitizer TBMPEI with AIE properties was synthesized *via* a new acceptor, and it was utilized as a necroptosis inducer during imaging-guided photodynamic therapy.

## Graphical abstract

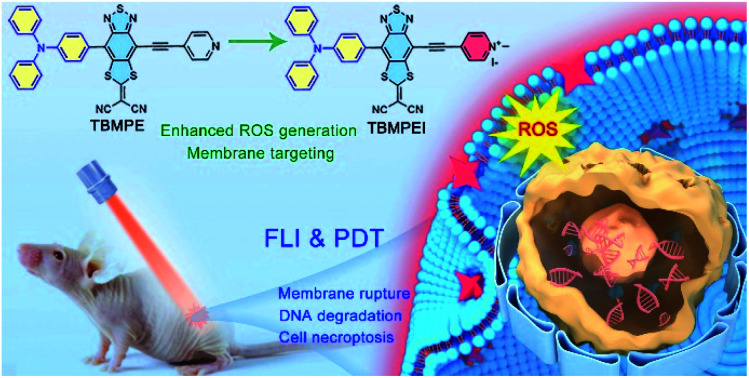

## Introduction

With the continuously increasing mortality rate caused by various cancers, exploring effective treatment strategies involving advanced technologies and/or materials remains a vital and urgent task worldwide.^1–5^ As a relatively new cancer treatment method, photodynamic therapy (PDT) has attracted a great deal of attention from both scientific researchers and clinicians by virtue of its distinctive advantages such as minimal invasiveness, high spatiotemporal precision, accurate controllability and insignificant side effects.^4,6–9^ In the PDT process, photosensitizers (PSs) can be excited upon light irradiation and undergo electron transfer and/or energy transfer to produce destructive reactive oxygen species (ROS) for ablating the exposed tumors by means of cell apoptosis or necrosis, vascular damage, and probably the immune response.^10,11^ However, the practical applications of PDT are generally limited by the insufficient lifetime (0.03–0.18 ms) and work span (0.01–0.02 μm) of ROS,^12,13^ and thus the photodynamic damage could only occur close to the location of the PS.^4^ Therefore, the location of the photosensitizer is critical to the PDT efficiency,^14–16^ and exploring critical site-specific targeting PSs is of significance for high-performance PDT.

In eukaryote cells, the biological functions are intimately associated with their various cell organelles.^17^ Among these organelles, the cell membrane serves as the “city wall” of the whole cell, protects cells from the outside, delivers nutrients/waste into/out of the cell and facilitates communication with other cells.^18^ Therefore, the cell membrane has been recognized as one of the most critical cell organelles and taken as one of the favoured targets for cancer treatment.^19–21^ The main structure of the cell membrane is composed of phospholipids, glycoproteins, glycolipids and proteins, and the phospholipid bilayer serves as the skeleton of the cell membrane,^18^ giving the cell membrane amphipathic properties and negative charges.^21^ To date, many cell membrane-targeting fluorophores have been exploited;^21–27^ however their applications towards fluorescence imaging (FLI)-guided PDT were largely restricted due to the respective and collective drawbacks including inferior imaging contrast, small Stokes shifts, severe photobleaching, and insufficient ROS production. The outcomes mainly result from the large co-planar π-conjugation structure of those conventional fluorophores, which usually exhibit attenuated fluorescence intensity and photosensitizing properties in aggregates or higher concentrations owing to the π–π stacking interaction, known as the aggregation-caused quenching effect.^28–31^ Given the circumstances, luminogens with aggregation-induced emission (AIE) features could be an ideal alternative to dramatically tackle these problems. AIE refers to a unique photophysical phenomenon that a family of luminogens are non-emissive in a molecularly dissolved state but the emission is dramatically boosted in aggregates.^32,33^ In addition, the ROS generation ability of AIEgens could be also promoted in the aggregated form.^34–36^ Besides, AIEgens generally feature large stokes shifts, which endows them with a high signal-to-noise fluorescence image. Hence, exploring distinctive AIEgen derived photosensitizers with cell membrane-specific anchoring capability would be significantly important.^37–40^

Additionally, the nucleus with large amounts of DNA is regarded as the “brain” of the cell, and hence a nucleus-targeting photosensitizer could trigger a more effective outcome than that in the cytoplasm.^14^ And the level of DNA damage during or after treatment has a tremendous impact on the treatment outcome.^41^ For example, the clinical success of cancer radiation therapy was limited by insufficient DNA damage.^42,43^ However, the nucleus is separated by the nuclear bilayer membrane with several pore complexes, and a conventional photosensitizer is difficult to get into the brain of the cell and cause direct DNA damage.^44^ Traditional lysosome or mitochondria targeting PSs usually induced cell apoptosis, where the DNA damage was not rapid and obvious.^15^ Hence, we suggest that we could find a cell membrane targeting photosensitizer; though it cannot enter into the cell nucleus, it could induce non-apoptotic cell death and indirectly have an influence on the integrity of the DNA and an effective anticancer effect.^17,22,39,43^ Herein, the molecular design would contain three elements: (1) rotatable units, (2) strong donor–acceptor (D–A) structure, and (3) positive charges, which was employed to meet the demands of AIE, photosensitizing ability and membrane binding ability, respectively.

**Scheme 1:**
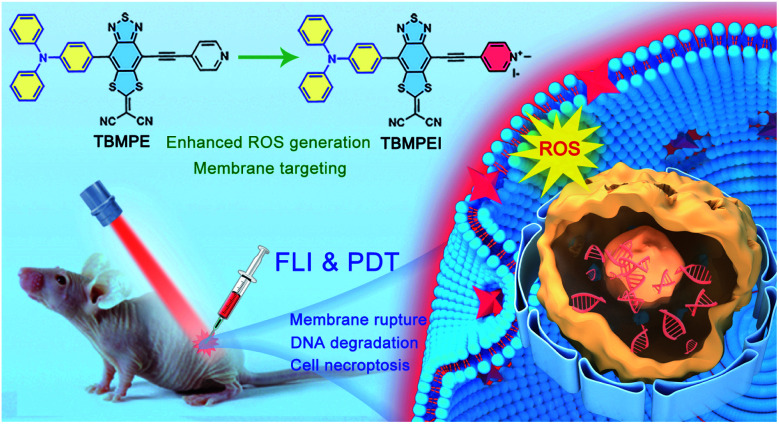
Schematic illustration of molecular design on a high-performance AIE photosensitizer with cell membrane-targeting function for fluorescence imaging-guided photodynamic cancer therapy.

Based on these considerations, the molecular structure in Scheme 1 was adopted. Triphenylamine (TPA) was chosen as the rotor and electron donor segment, and a novel electron acceptor 2-(4-methyl-8-(pyridin-4-ylethynyl)[1,3]dithiolo[4′,5′:4,5]benzo[1,2*c*][1,2,5]thiadiazol-6-ylidene)malononitrile was utilized as a new strong acceptor to achieve near-infrared (NIR) emission. To target the cell membrane and enhance the photosensitizing ability, the pyridine unit was further cationized into a pyridine salt. Hence, two AIEgens, namely non-cationic TBMPE and cationic TBMPEI, were obtained. These AIEgens exhibited a broad absorption band in the whole visible light range and NIR fluorescence emission. Meanwhile, the ROS generation capability of TBMPEI was far superior to those of popularly used photosensitizers. Importantly, TBMPEI was able to selectively accumulate on the cell membrane and induce cell necroptosis by light irradiation, accompanied by membrane rupture and DNA degradation. *In vivo* evaluation showed that TBMPEI is an excellent candidate for fluorescence imaging guided PDT.

## Results and discussion

### Synthesis and photophysical properties

**Fig. 1:**
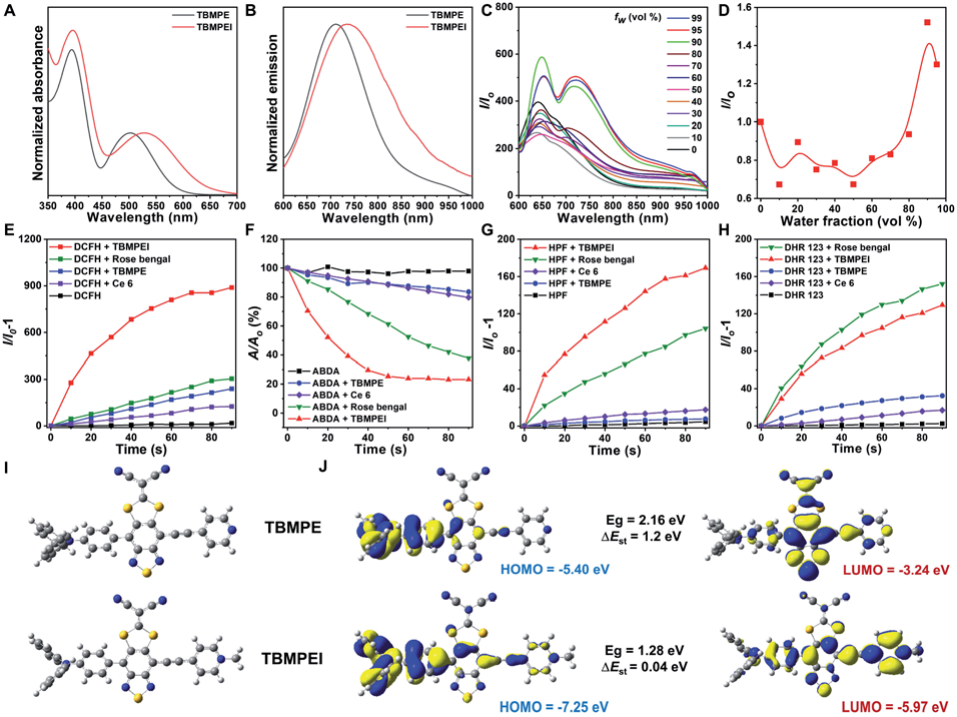
Normalized (A) absorption and (B) emission spectra of TBMPE and TBMPEI dissolved in THF. (C) Fluorescence spectra of TBMPEI in a DMSO/H_2_O mixture with different water fractions. (D) Plots of the relative PL intensity (*I*/*I*_0_) of TBMPEI *versus* water fraction. (E) ROS generation of various molecules upon white LED lamp irradiation using DCFH as the indicator. ROS differentiation of these molecules by using (F) ABDA for ^1^O_2_, (G) HPF for ˙OH and (H) DHR 123 for O_2_^−^ as the indicator. (I) Optimized structures of TBMPE and TBMPEI. (J) Molecular orbital amplitude plots of the HOMO and LUMO energy levels of TBMPE and TBMPEI. Calculations were performed by DFT theory calculations at the m062x/6-31g* level using the Gaussian 09 program.

The detailed synthetic procedures are given in Scheme S1.† 4,7-Dibromo-5,6-difluorobenzo[*c*][1,2,5]thiadiazole reacted with (4-(diphenylamino)phenyl)boronic acid to produce a donor–acceptor intermediate, which further coupled with 4-ethynylpyridine. Later, it reacted with sodium 2,2-dicyanoethene-1,1-bis(thiolate) and TBMPE was obtained.^45^ TBMPEI was produced through TBMPE cationized by iodomethane. The structures of the products are well characterized in Fig. S1–S6† in the ESI. The photophysical properties of these two compounds were investigated by using UV-vis absorption and fluorescence spectra. As depicted in Fig. 1A, both TBMPE and TBMPEI possess broad absorption in the visible light range from 400 to 700 nm. TBMPE showed two absorption peaks around 390 and 500 nm in THF solution, in which the first one resulted from the local excited state, and the latter one was ascribed to charge transfer. In the case of TBMPEI, two absorption peaks were located at 390 and 530 nm. The red shift of the second absorption peak could be attributed to the stronger charge transfer properties of TBMPEI. As shown in Fig. 1B, TBMPE and TBMPEI exhibited emission peaks at ∼710 and ∼735 nm, respectively. To inspect their AIE characteristics, mixed solutions (DMSO/H_2_O) with different water fractions were employed. As illustrated in Fig. 1C and D, the fluorescence intensity of TBMPEI initially decreased with the increase of the H_2_O fraction, which arose from the twisted intramolecular charge transfer (TICT) effect. A quick increase was then observed with the water fraction increased over 50%, and the maximum emission intensity was reached at the 95% water fraction, indicating the typical AIE properties.^46^ The fluorescence spectra of TBMPE had a similar tendency with the increase of water fractions (Fig. S7†). Moreover, the emission spectra of TBMPE and TBMPEI in the solid state were centred at 732 and 778 nm, respectively (Fig. S8†). Notably, the large Stokes shift (>150 nm) and NIR emission of these two AIEgens could efficiently avoid the background noise during fluorescence imaging.^47^

The ROS generation ability of these two AIEgens was then assessed by using DCFH as the indicator, which is non-emissive in the natural state but the emission can be largely boosted in the presence of ROS.^48^ As shown in Fig. 1E and S9,† with the prolonged time of white light irradiation, the fluorescence signals of DCFH incubated with TBMPE or TBMPEI continuously increased, while pure DCFH showed negligible fluorescence enhancement. In addition, TBMPEI presented far better performance than TBMPE, as well as commercially available Rose bengal and Ce6. Furthermore, the discrimination of ROS generated by TBMPEI was conducted using various commercial indicators, such as ABDA absorption for ^1^O_2_, HPF fluorescence for ˙OH and DHR 123 fluorescence for O_2_^−^. It was observed that the absorption of ABDA showed an obvious decrease in the presence of TBMPEI and light irradiation, and the fluorescence signal of HPF and DHR 123 increased, denoting that the ROS generated by TBMPEI were a mixture of type I (˙OH, O_2_^−^) and type II (^1^O_2_) ROS (Fig. 1F–H and S10–S12†).

To better understand the photophysical properties of these AIEgens, density functional theory (DFT) calculations were performed at the m062x/6-31g level with molecular geometries optimized at the m062x/6-31g* level. As demonstrated in Fig. 1I and J, a more obvious separation of HOMO and LUMO distribution was detected in the case of TBMPEI than that of TBMPE. TBMPEI's HOMO–LUMO energy gap is smaller than that of TBMPE, which could explain the longer absorption wavelength of TBMPEI. Additionally, the energy gap between the S1 and T1 states (Δ*E*_st_) of these two molecules was calculated (Fig. S13†). A smaller Δ*E*_st_ of ∼0.04 eV was calculated for TBMPEI, which is well consistent with the previously obtained photo-physical properties, demonstrating that the induction of a cationic pyridine structure could efficiently enhance ROS generation, making TBMPEI an excellent AIE photosensitizer.

### Membrane-targeting ability

**Fig. 2:**
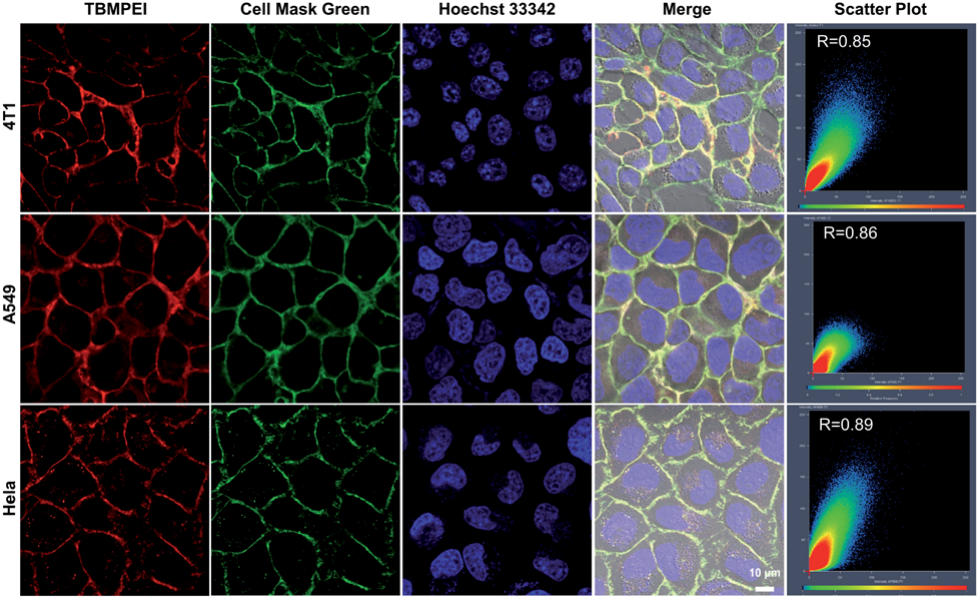
CLSM images of 4T1, A549 and HeLa cells co-incubated with TBMPEI (10 μM, 30 min), Cell Mask Green (1 μM, 30 min) and Hoechst 33342 (1 μM, 30 min) and relative overlapping coefficient assessed from the Pearson correlation coefficient. (TBMPEI, Ex: 488 nm, Em: 600–700 nm; Cell Mask Green, Ex: 488 nm, Em: 500–590 nm; Hoechst 33342, Ex: 405 nm, Em: 430–470 nm). Scale bar: 10 μm.

The cellular uptake and cellular location are important for the photosensitizer's theranostic efficiency.^49^ In the preliminary study, the uptake and distribution of TBMPEI were evaluated by using 4T1 cells as the model cancer cell line. As shown in Fig. S14,† after 15 min incubation, the cells showed bright red fluorescence around the boundary of cells, denoting that TBMPEI efficiently bound and stained the cell membrane structure. The influence of the incubation period was then investigated with different staining times (15 min to 1 h). The results demonstrated that incubation for 15 min and 1 h showed no obvious fluorescence imaging quality change. Furthermore, the cell membrane-specific targeting performance was confirmed by co-staining with nucleic dye Hoechst 33342 and plasma membrane dye Cell Mask Green. Moreover, a variety of cancer cell lines, including breast cancer cells 4T1, lung cancer cells A549 and cervical carcinoma cells HeLa, were employed to investigate the cell labelling efficiency. As depicted in Fig. 2, the green fluorescence of Cell Mask Green overlapped well with the red fluorescence of TBMPEI, and the Pearson coefficients between these two dyes were determined to be 0.85, 0.86 and 0.89, respectively. In all tested cases, the plasma membrane was clearly visualized with a high signal-to-noise ratio of cell imaging with intensive red emission, suggesting the excellent applicability of TBMPEI to various cell types. In addition, after the incubation of TBMPE for 30 min, bright fluorescence dots were observed inside the cells, and a good overlap with commercial dye lysosome blue was observed (Fig. S15†). The results demonstrated that positive charge has a significant influence on the intracellular location. Moreover, to assess the photostability of TBMPEI, continuous excitation and sequential scanning with a confocal microscope were performed, and Cell Mask Green was chosen as the control as shown in Fig. S16.† The result showed that the fluorescence signal of TBMPEI remained bright during 50 loops of irradiation, and the fluorescence loss of Cell Mask Green was more evident under the same conditions, solidly suggesting the excellent photostability of TBMPEI.

### Cytotoxicity and induced cell death pathway

**Fig. 3:**
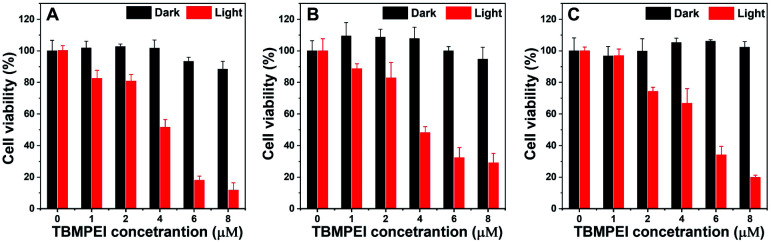
Cell viability of (A) 4T1, (B) A549 and (C) HeLa cells treated with various amounts of TBMPEI without or with light irradiation (white LED lamp, 24 mW cm^−2^, and 10 min).

**Fig. 4:**
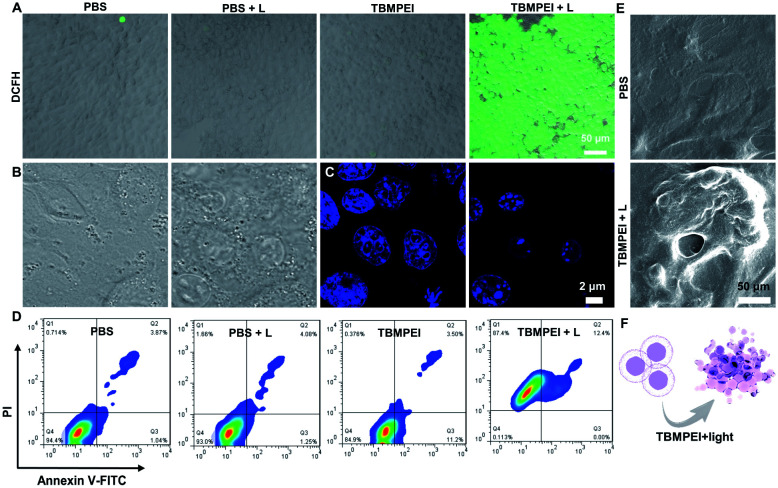
(A) CLSM images of 4T1 cells stained with DCFH-DA (10 μM) after various treatments (PBS, PBS + Light, TBMPEI, and TBMPEI + Light). Scale bar: 50 μm. (B) Morphological changes of 4T1 cells stained with TBMPEI (10 μM) under continuous 488 nm laser irradiation and corresponding (C) nucleic acid change stained with Hoechst 33342 (1 μM). (D) Apoptosis analysis of 4T1 cells induced by various treatments using an Annexin V-FITC/PI assay kit by flow cytometry. Q1, Q2, Q3, and Q4 represent necrotic, late apoptotic, early apoptotic, and normal cells, respectively. (E) Morphological features of normal and TBMPEI + light treated cells revealed by scanning electron microscopy. (F) Illustration of the cell necroptosis pathway induced by TBMPEI + light treatment.

Inspired by its extraordinary ROS generation capability and cell membrane-targeting behaviour, TBMPEI was utilized to ablate the cancer cells by means of PDT. The cytotoxicity of TBMPEI was evaluated *via* a traditional cell counting kit-8 (CCK 8) assay.^50^ 4T1, A549 and HeLa cells were respectively incubated with different amounts of TBMPEI and further treated without/with white light irradiation. As shown in Fig. 3, the dark toxicity of TBMPEI towards all three cell lines could be neglected in the tested range. However, the light toxicity of TBMPEI against all three cell lines was significant. And the IC_50_ value of TBMPEI upon light irradiation on 4T1, A549 and HeLa cells was calculated to be 3.63, 4.17 and 4.55 μM, respectively. These results demonstrated that TBMPEI could achieve effective cell ablation. Additionally, the toxicity of TBMPE without/with light irradiation was also measured by using a CCK-8 assay as shown in Fig. S17.† Although TBMPE exhibited a certain ROS generation ability *in vitro*, no apparent toxicity was observed under the same conditions. Then more detailed information about cell death induced by TBMPEI was obtained by taking 4T1 cells as the model cell. Firstly, the intracellular ROS generation of TBMPEI upon light irradiation was evaluated by utilizing the indicator DCFH-DA.^51^ As illustrated in Fig. 4A, 4T1 cells in control experiments (PBS, PBS + light, and TBMPEI) showed negligible fluorescence in the whole image, and the cell treated with TBMPEI plus light exhibited bright green fluorescence upon irradiation. The results denoted that the incubation of TBMPEI and irradiation with light could result in ROS generation. To further verify that the ROS generation is the key criterion to induce cell death, the intracellular lipid peroxidation after treatment was detected by measuring malondialdehyde (MDA), a natural product of lipid oxidization.^51^ As demonstrated in Fig. S18,† the MDA content of cells treated with TBMPEI plus light irradiation has a ∼7.2 fold increase in comparison with the cells treated with PBS. For other control groups (cells + light and cells + TBMPEI), no obvious MDA increase was observed. Those outcomes strongly demonstrated that the cytotoxicity of TBMPEI was certainly derived from ROS generation. The morphological change of cells during light irradiation was also observed in real time under a CLSM by using a 488 nm laser as the light source. The commercially available nuclear dye Hoechst 33342 was used as a co-staining dye to locate the positions of the cells and observe the nuclear change. The duration of light irradiation was about 6 min with 60 loops. As shown in ESI movies S1–S3† and Fig. 4B and C, the treated cells swelled and their size increased, and then the nuclear structure also shrank. After 60 loops of irradiation, the integrity of the cell membrane was totally lost, and the degradation of DNA was observed. To avoid the laser toxicity during the continuous light irradiation, cells stained with Hoechst 33342 were used as the control experiment (ESI movie S4 and Fig. S19†). Differently, there was no obvious fluorescence signal decrease, neither clumping or degradation of DNA. Furthermore, a white LED lamp was used as another light resource to observe the cell morphology change during the treatment. As demonstrated in Fig. S20,† the cell membrane structure was totally damaged, and the degradation of DNA was also detected. Additionally, during the light irradiation, the fluorescence signal of TBMPEI was retained in the region of the cytoplasm (ESI movie S3†), and thus DNA damage of ROS generated by TBMPEI could be ignored.

All these observed characteristics were totally different from classical apoptosis, which is denoted by cell shrinkage, condensation of chromatin and an intact cell membrane.^52,53^ To validate the hypothesis that TBMPEI induced cell non-apoptosis death, cells were further investigated by Annexin V-FITC (Fluorescein Isothiocyanate)/PI (Propidium Iodide) double staining to evaluate the ratio of apoptotic cells after various treatment. Annexin V^+^/PI^−^ is usually defined as apoptotic cells, and Annexin V^+^/PI^+^ cells were denied as necrotic cells.^54,55^ As shown in Fig. 4D, both apoptosis and necrosis were induced during TBMPEI plus light therapy, and the majority of cells were necrotic cells (about 87.4% for necrotic and 12.4% for apoptotic). In contrast, PBS and PBS plus light induced only about ∼6% apoptotic/necrotic cells, and TBMPEI induced about ∼15% apoptotic/necrotic cells. The flow cytometry results were also consistent with the FDA/PI double staining results in Fig. S21.† The morphological change of 4T1 cells after TBMPEI plus light treatment was also characterized by using a scanning electron microscope (Fig. 4E). Additionally, to evaluate the integrity of the cell membrane, the LDH release after treatment was also compared. As collected in Fig. S22,† after treatment with TBMPEI plus light, the release of LDH into the medium showed a ∼2 fold enhancement. Based on these results, we suggested that the membrane-targeting photosensitizer TBMPEI could induce a fast necroptosis cell death pathway. More importantly, the degradation of DNA during treatment could effectively hamper the cell division or tumor invasion.^56^

### *In vivo* fluorescence imaging guided therapy

**Fig. 5:**
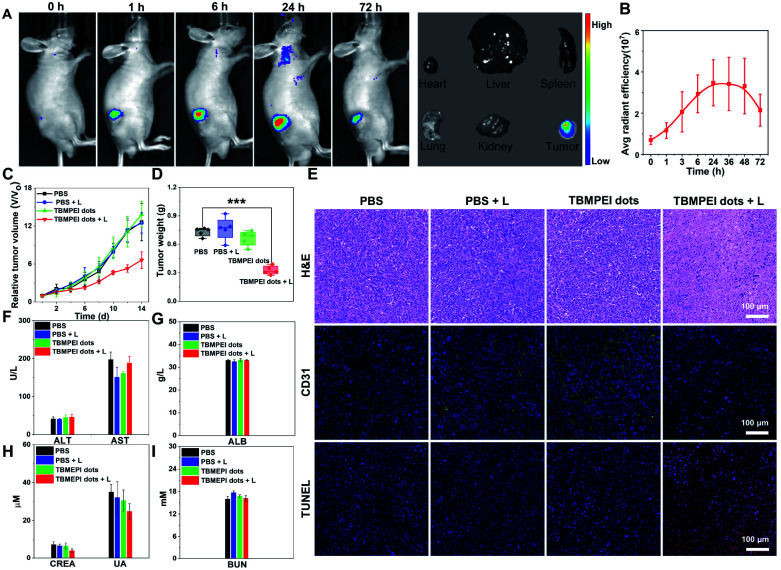
(A) Fluorescence images of tumor-bearing mice at different time points after the injection of TBMPEI dots, and *ex vivo* fluorescence images of tumors and major organs after the injection of TBMPEI dots for 72 h. (B) Corresponding average radiant efficiency of tumor-bearing mice at different time points after the injection of TBMPEI dots. (C) Tumor growth curves of mice with different treatments. (D) Tumor weight of mice in different groups after 14 days of treatment. (E) H&E, CD31 and TUNEL staining analyses of tumor tissues in different groups after 14 days of treatment. Scale bar: 100 μm. (F–I) Blood biochemistry and hematology data of mice after different treatments.

Based on the above excellent imaging and therapeutic properties of TBMPEI *in vitro*, the *in vivo* imaging-guided therapy ability was investigated by taking 4T1 tumor-bearing mice as animal mode. To facilitate its application for bio-systems, water-soluble TBMPEI dots were prepared *via* self-assembly.^57^ The successful formation of dots was characterized *via* TEM and DLS as shown in Fig. S23.† The TBMPEI dots were intratumorally injected into the tumor, and the relative fluorescence images were collected at various time points. As depicted in Fig. 5A, the fluorescence signal of TBMPEI was clearly observed at the tumor site upon injection. The fluorescence signal showed a slight increase within 6–24 h, which is supposed to be caused by the gradual diffusion and penetration of TBMPEI dots. The high fluorescence signal was retained for a long time, and a noticeable decrease after 72 h was observed. To further investigate the bio-distribution of TBMPEI dots, the mice were sacrificed after 72 h, and the tumor and main organs were harvested and imaged. It was found that the fluorescence signal was mainly located in the tumor tissues. The quantification of TBMPEI dot fluorescence at tumor sites is collected in Fig. 5B; the fluorescence intensity of TBMPEI at tumor sites increased (6–24 h), and then decreased slowly (48–72 h). Besides, mice after 24 h injection were sacrificed and the major organs were imaged as shown in Fig. S24,† and the fluorescence signal was also observed in the liver and kidney region. These results demonstrate that the intratumor injection of TBMPEI dots showed an ultra-long retention time in the tumor region, and the dots may be slowly metabolized through the liver and kidney.^58^

After confirming the *in vivo* imaging ability of TBMPEI, the *in vivo* antitumor performance of TBMPEI dots was also assessed. The same tumor model was employed, and PBS, PBS plus light, and TBMPEI dots were taken as the control experiments. White light irradiation (400–700 nm and 100 mW cm^−2^) was performed for 20 min after 24 h injection. The tumor size was measured every 2 days during the 14 day treatment and the relative growth curve was collected. The tumor volumes in control groups showed an obvious increase during the period, while the group treated with TBMPEI dots plus light irradiation showed a pronounced suppression effect on the tumor growth (Fig. 5C and S25†). After 14 days of treatment, all mice were sacrificed, and the weight of the tumors was measured. As shown in Fig. 5D, a tumor inhibition rate of 65% was obtained after 14 days of treatment of TBMPEI dots, demonstrating a good photodynamic therapeutic effect. To further explore the therapeutic mechanism, dissected tumors were further examined by histological (hematoxylin and eosin staining, H&E staining) and immunohistochemical (TUNEL and CD31) analysis (Fig. 5E). For the control groups (PBS, PBS + light, and TBMPEI dots), abundant and densely arranged cells with intact cell plasma were observed in the tumors. In contrast, for the experimental group (TBMPEI dots + light), obvious abnormal cells with void cell plasma and shrinkaged cell nuclear were detected in the tumor section. TUNEL immunofluorescence results also confirmed that the treatment of TBMPEI dots with light irradiation induced a number of apoptotic cells in the tumor site, while no apparent apoptotic cell was observed in other control groups. Additionally, only a few CD31 positive new micro-vessels were visualized in the TBMPEI dots plus light group, which is massive in other control groups. The results were also in accordance with the TUNEL immunofluorescence results. Additionally, the weights of mice in all treated groups have a gradual increase (Fig. S26†), suggesting that the treatment of both TBMPEI dots and light irradiation caused insignificant systemic toxicity.

To investigate the biosafety of TBMPEI dots during phototherapy, the blood biochemistry and hematology data of treated Balb/c mice were collected. As shown in Fig. 5F–I, the levels of alanine aminotransferase (ALT), aspartate aminotransferase (AST) and albumin (ALB), which are liver function indices, and the levels of creatinine (CREA), uric acid (UA) and urea nitrogen (BUN), which are kidney function indices, were all in the normal ranges and almost unchanged in comparison with the control groups. Furthermore, no distinct abnormalities were observed in the H&E staining results of the major organs of mice after 14 days of treatment (Fig. S27†). In the consideration of the positive charge of TBMPEI, the hemolysis assay was performed *in vitro* to evaluate its safety. As depicted in Fig. S28,† in the whole range (1–10 μM), no obvious hemolytic phenomenon was observed, and the rate of hemolysis remained <5%, denoting that the TBMPEI showed a negligible hemolysis effect. Based on all these results, it was demonstrated that TBMPEI could serve as a safe, appropriate and efficient photosensitizer for cancer imaging and ablation.

## Conclusions

In summary, we developed two novel NIR AIEgens on the basis of a new electron acceptor moiety. One of these AIEgens, TBMPEI, exhibits high ROS generation efficiency even far superior to some popularly used and reputable photosensitizers, as well as cell membrane-specific targeting capability towards cancer cells. An *in vitro* test demonstrates that TBMPEI is capable of ablating cancer cells upon light irradiation through cell necroptosis with cell membrane rupture and DNA degradation. Moreover, TBMPEI also well performs for *in vivo* fluorescence imaging-guided photodynamic therapy. This study thus provides a promising design strategy for exploring advanced theranostic agents for cancer treatment.

## Experimental section

### Materials

All reagents were commercially available and used as supplied without further purification. Organic solvents were either employed as purchased or dried according to procedures described in the literature.

### Characterization

NMR spectra were recorded on a Bruker ARX 400 NMR spectrometer using CDCl_3_ and tetramethylsilane (TMS; *δ* = 0 ppm) as the internal reference. High-resolution mass spectroscopy (HRMS) was carried out on a GCT premier CAB048 mass spectrophotometer operating in MALDI-TOF mode. UV/vis absorption spectra were recorded on a Shimadzu 2550 UV/vis spectrophotometer. The photoluminescence (PL) spectra were recorded on a Horiba Fluorolog-3 spectrofluorometer.

### Synthesis of TBMPE and TBMPEI

TBMPE was synthesized according to procedures described in the literature.^45^ TBMPEI : TBMPE (124 mg, 0.2 mmol) was dissolved in 30 mL acetone, and then iodomethane (141 mg, 1 mmol) was added into it. The reaction mixture was heated to reflux for 48 h under a N_2_ atmosphere. After the reaction, the solvent was removed, and the final product (150 mg) was obtained. ^1^H NMR (400 MHz, CDCl_3_, 298 K) *δ* (ppm): 9.38–9.36 (2H, d, *J* = 8 Hz), 8.22–8.21 (2H, d, *J* = 4 Hz), 7.54–7.52 (2H, d, *J* = 8 Hz), 7.39–7.35 (4H, m), 7.24–7.16 (4H, m), 7.16–7.14 (4H, m), 4.75 (3H, s). ^13^C NMR (100 MHz, CDCl_3_, 298 K) *δ* (ppm): 176.08, 153.45, 152.39, 150.32, 146.31, 145.91, 144.22, 138.26, 135.44, 132.49, 130.20, 129.79, 129.69, 126.25, 125.65, 124.91, 120.15, 111.90, 105.58, 96.99. HRMS: *m*/*z* calculated for 633.0984; found at 633.1029.

### Detection of ROS generation

The total ROS generation ability of these compounds was firstly investigated *via* the fluorescence of DCFH. To prepare DCFH solution, 0.5 mL of DCFH-DA (1 mM) was added to 2 mL of NaOH (10 mM) solution and hydrolyzed for 30 min at room temperature. Then the solution was neutralized with 10 mL PBS (pH = 7.4) and a DCFH stock solution (40 μM) was obtained and kept in the dark for further usage. Afterwards, mixtures of 750 μL of DCFH, 2247 μL of PBS, and 3 μL of TBMPE (TBMPEI, Rose Bengal and Ce 6) were prepared and irradiated with a white lamp for different time periods. The fluorescence spectra at different time periods were collected with excitation of 488 nm.

To verify the ROS species, the commercial ROS indicators ABDA, HPF and DHR 123 were used as specific indicators to distinguish singlet oxygen (^1^O_2_), hydroxyl radicals (˙OH) and superoxide anions (O_2_^−^). For ˙OH and O_2_^−^ detection, stock solutions of these indicators (HPF and DHR 123, 1 mM) were prepared, and then 30 μL of these indicators was mixed with 6 μL of photosensitizer (1 mM) in 3 mL PBS, respectively. For ^1^O_2_ detection, a stock solution of ABDA (5 mM) was prepared, and then 12 μL of indicator was mixed with 6 μL of photosensitizer (1 mM) in 3 mL PBS. Finally, the mixtures were irradiated with a white lamp for different time periods and their corresponding absorption and fluorescence spectra were collected. The pure indicators in the PBS were chosen as the control experiments.

### Cell culture and cell imaging

The 4T1 cells were cultured in a 1640 culture medium containing 10% FBS and 1% penicillin-streptomycin at 37 °C in a humidified environment of 5% CO_2_. The A549 cells and HeLa cells were cultured in a DMEM culture medium containing 10% FBS and 1% penicillin-streptomycin under the same conditions.

For cellular uptake experiments, 4T1 cells were seeded into confocal dishes with a glass bottom and cultured for 36 h. Then the old culture medium was removed and 1 mL fresh medium containing 10 μM TBMPEI was added. After various incubation times (15 min, 30 min and 1 h), the cells were washed with PBS twice and observed under a confocal laser scanning microscope (CLSM). Excitation: 488 nm. Emission filter: 600–700 nm.

For the co-staining experiment, 4T1 cells, A549 cells and HeLa cells were seeded into confocal dishes and cultured in an incubator. After 24 h incubation, the old culture medium was removed and 1 mL of fresh medium containing 10 μM TBMPEI, 1 μM Cell Mask Green and 1 μM Hoechst 33342 was added. After 30 min incubation, the cells were rinsed with PBS 3 times, and then imaged under a CLSM. Excitation wavelength: 405 nm for Hoechst 33342 and 488 nm for TBMPEI and Cell Mask Green. Emission filter: 410–500 nm for Hoechst 33342, 500–590 nm for Cell Mask Green, and 600–700 nm for TBMPEI.

### Cytotoxicity test

The cytotoxicity of TBMPEI towards three cancer cell lines was investigated by using a CCK-8 assay kit. In brief, 100 μL culture medium containing 5 × 10^3^ cells was added into each well of a 96-well plate and grown overnight. Then the old medium was removed and fresh medium containing various amounts of TBMPEI was added into each cell. After 3 hours of incubation, the light treated groups were irradiated with a white LED lamp for 10 min. Subsequently, the cells were incubated for 21 h. Finally, the medium of each well was removed and replaced by 100 μL fresh medium containing 10% CCK solution and incubated for 1 h. Finally, the absorbance at 450 nm was measured by using a microplate reader and the relative cell viability was calculated by using the following equation:Cell viability (%) = (OD_sample_ − OD_background_)/(OD_control_ − OD_background_) × 100%.

### Intracellular ROS generation

To measure intracellular ROS generation, a commercially available ROS detection kit was employed according to the manufacturer's instructions and DCFH-DA was used as the indicator. Briefly, 4T1 cells were seeded into a 6-well plate and grown for 24 h. The old medium was replaced with a fresh medium with/without TBMPEI for 3 h. Then the cells were washed with PBS 3 times, and incubated with an FBS-free medium, containing 10 μM DCFH-DA for another 30 min. For the TBMPEI + light/PBS + light groups, the cells were irradiated with a white LED lamp for 10 min, and then incubated for another 30 min at 37 °C. For TBMPEI/PBS groups, the cells were merely incubated at 37 °C. After final incubation, the images of cells were collected with excitation at 488 nm and emission from 500–550 nm.

### Intracellular MDA content detection

Typically, 4T1 cells were seeded into 6 cm dishes and grown for 24 h. Then the cells were incubated with fresh medium with/without TBMPEI (10 μM) for another 3 h. Subsequently, the cells were irradiated with a white LED lamp for 10 min and further incubated 3 h. After incubation, the cells were washed and harvested by trypsinization. Cellular extracts were prepared with an ultrasonic disruptor and then the lysed cells were centrifuged at 12 000 rpm for 5 min to remove the sediment.

To detect the amount of MDA, 100 μL supernatant was mixed with 200 μL TBA detection solution and incubated at 100 °C for 15 min. After cooling to room temperature, the mixtures were centrifuged at 1200 rpm for 10 min. Then the obtained 200 μL supernatant was added to a 96-well plate, and the absorbance at 540 nm was read *via* a microplate. The amounts of MDA were calculated from the obtained standard curves.

To detect the amount of protein, 20 μL supernatant was mixed with 200 μL BCA detection solution and incubated at room temperature for 1 h. Then, the mixture was added into a 96-well plate, and the absorbance at 570 nm was read *via* a microplate. The amounts of MDA were calculated from the obtained standard curves.

### Apoptosis/necrosis detection by flow cytometry

Harvested 4T1 cells (10^5^ cells per well) were seeded and cultured in 6-well plates for 24 h. Then, the cells were treated with TBMPEI (10 μM) for 3 h, followed by white light irradiation (400–700 nm and 24 mW cm^−2^) for 10 min. After 12 h of incubation, the cells were carefully collected and washed with PBS three times by centrifugation (1000 rpm, 5 min, and 4 °C). The samples were then stained with an Annexin V-FITC/PI Apoptosis Detection Kit according to the manufacturer's instructions and analyzed by flow cytometry.

### LDH release

100 μL culture medium containing 1 × 10^4^ cells was added into each well of a 96-well plate and incubated overnight. Then the cells were treated with 0, 5, 10 and 20 μM TBMPEI for 3 h, and the cells were washed with PBS and 100 μL of fresh medium without FBS were added into the well. Then light irradiation was performed with white light (24 mW cm^−2^ and 10 min), and the cells were incubated for another 3 h for the release of LDH. Finally, the medium of each well was collected, and the LDH release was measured according to the manufacturer's guide, and then the absorbance at 490 nm was recorded. For the control groups, cells were incubated with various amounts of TBMPEI for the same time without light irradiation, and for the negative group, the cells were incubated with a LDH inducer for 30 min, and the supernatant was collected and detected, and medium (no cells and no FBS) was chosen as the negative groups.

### Animals and tumor model

Healthy BALB/c nude mice (male, 4–5 weeks) were obtained from Beijing Vital River Laboratory Animal Technology. The mice were housed under pathogen-free conditions and fed with standard laboratory water and chow. A xenograft 4T1 tumor-bearing mice model was established through subcutaneous injection of 4T1 cells (5 × 10^5^) suspended in PBS into the right flank of mice. The 4T1 tumor-bearing mice were subsequently used when the tumor volumes reached about 100 mm^3^. The experiment was performed in strict accordance with the guidelines of the Institutional Animal Care and Use Committee of China, and was approved by the Animal Ethical and Welfare Committee of Shenzhen Graduate School, Peking University (Shenzhen, China).

### *In vivo* fluorescence imaging

To investigate the *in vivo* fluorescence imaging ability of TBMPEI dots, the xenograft 4T1 tumor-bearing mice were selected as the 4T1 tumor-bearing mice and intratumorally injected with 20 μL TBMPEI dots, and then images at 1, 3, 6, 24, 36, 48, and 72 h were collected by using an IVIS Spectrum imaging system (Exi: 465 nm and Emi: 780 nm). After 72 h, the mice were subsequently sacrificed, and the major organs (heart, liver, spleen, lung and kidney) and tumor were collected. The fluorescence picture of the major organs and tumor was also collected using the same conditions. The related quantitative analyses of intensity were performed using the IVIS Spectrum imaging system.

### *In vivo* photodynamic therapy

To investigate the *in vivo* antitumor efficacy of TBMPEI dots, the xenograft 4T1 tumor-bearing mice were divided into 4 groups (*n* = 5, including PBS, PBS + light, TBMPEI dots, and TBMPEI dots + light). When the volume of the tumors reached ∼100 mm^3^, the mice were administered with 20 μL PBS and TBMPEI dots *via* intratumoral injection. For the PBS + light and TBMPEI dots + light groups, after 24 h injection, the tumors of mice in each group were continuously irradiated with a white LED lamp (100 mW cm^−2^) for 20 min. The treatment was repeated every 2 days. After various treatments, the tumor size was measured by using a vernier caliper, and the weights of mice were also recorded. The tumor size was estimated using the following formula:*V* = (length × width^2^)/2

### Histological and hematological analyses

After complete treatment of 14 days, all the mice were humanely sacrificed, followed by the excision of tumors. The tumors were weighed and a picture was taken, and they were then fixed in 4% paraformaldehyde overnight, embedded in paraffin, and sliced at 5 μm thickness. Then the obtained paraffin section was stained with H&E staining, immunohistochemical TUNEL and CD31 staining. Finally, the stained slices were imaged with an inverted optical microscope and fluorescence microscope, respectively. To investigate the biosafety of treatment, the major organs (heart, liver, spleen, lung, and kidney) of each mouse were also excised, and H&E staining was performed in the same procedures.

### Hemolytic assay

A whole blood sample (0.5 mL) was collected from the Balb/C mouse by enucleation of the eyeball, and then the fresh blood was diluted with 4.5 mL PBS, and then centrifuged at 3000 rpm for 5 min at 4 °C. The collected RBCs were further washed with PBS 3 times, and finally diluted to 5 mL PBS. Then 0.2 mL of diluted RBC was incubated with 0.8 mL of PBS containing various amounts of TBMPEI dots (the final concentration of TBMPEI dots was 1, 2, 5 and 10 μM) at 37 °C for 2 h. For the positive control, 0.2 mL of RBCs were incubated with 0.8 mL pure water, and for the negative control, 0.2 mL of RBCs were incubated with 0.8 mL pure water. After incubation, all samples were centrifuged at 3000 rpm for 5 min at 4 °C, and the picture was collected and the absorbance of the supernatant at 514 nm was read by using a microplate. The absorbance of TBMPEI dots at various concentrations was also detected. The percent of hemolysis was calculated as follows:Hemolysis (%) = (Abs_sample_ − Abs_negtive_ − Abs_TBMPEI_)/(Abs_positive_ − Abs_negative_) × 100%

## Data availability

All experimental supporting data and procedures are available in the ESI.†

## Author contributions

Niu Niu: investigation and writing–original draft; Ying Yu: synthesis, investigation, and writing–review & editing; Zhijun Zhang: investigation; Miaomiao Kang: investigation; Lei Wang: writing–review & editing; Zheng Zhao: supervision writing–review & editing; Dong Wang: supervision, methodology, and writing–review & editing. Ben Zhong Tang: supervision, methodology, and writing–review & editing.

## Conflicts of interest

There are no conflicts to declare.

## Supplementary Material

SC-013-D2SC01260J-s001

SC-013-D2SC01260J-s002

SC-013-D2SC01260J-s003

SC-013-D2SC01260J-s004

SC-013-D2SC01260J-s005
