# Supplementary material for: A cell membrane-targeting AIE photosensitizer as a necroptosis inducer for boosting cancer theranostics
Source: Chem Sci. 2022 Apr 19;13(20):5929–37. doi: 10.1039/d2sc01260j (PMC9132078; doi:10.1039/d2sc01260j)
Supplement: SC-013-D2SC01260J-s001 [file SC-013-D2SC01260J-s001.pdf]

Electronic Supplementary Material (ESI) for Chemical Science.
This journal is © The Royal Society of Chemistry 2022

Cell membrane-targeting AIE photosensitizer as necroptosis inducer for boosting cancer theranostics

Niu Niu,[†ab] Ying Yu,[†c] Zhijun Zhang,[ab] Miaomiao Kang,[ab] Lei Wang,[ab] Zhen Zhao,[*c] Dong Wang[*ab] and Ben Zhong Tang[*c]

Table of Contents

Scheme S1. Synthesis routine of TBMPE and TBMPEI. S1

Fig. S1 ^{1}H NMR spectrum of TBMPE in $CDCl_3$. S1

Fig. S2 ^{13}C NMR spectrum of TBMPE in $CDCl_3$. S2

Fig. S3 HRMS spectrum of TBMPE. S2

Fig. S4 ^{1}H NMR spectrum of TBMPEI in $CDCl_3$. S3

Fig. S5 ^{13}C NMR spectrum of TBMPEI in $CDCl_3$. S3

Fig. S6 HRMS spectrum of TBMPEI. S4

Fig. S7 AIE curve of TBMPE in THF/H_2O mixture. S4

Fig. S8 Absorption and emission spectra of TBMPE and TBMPEI in solid states. S4

Fig. S9 Fluorescence spectra change of DCFH with different photosensitizers. S5

Fig. S10 Absorption spectra change of ABDA with different photosensitizers. S5

Fig. S11 Fluorescence spectra change of HPF with different photosensitizers. S5

Fig. S12 Fluorescence spectra change of DHR 123 with different photosensitizers. S5

Fig. S13 DFT calculation of TBMPE and TBMPEI. S6

Fig. S14 CLSM image of cells stained for different time. S6

Fig. S15 CLSM image of cells stained with TBMPE. S6

Fig. S16 Photostability of TBMPEI. S7

Fig. S17 Cytotoxicity of TBMPE towards 4T1 cells. S7

Fig. S18 MDA release assay of 4T1 cells after different treatment. S7

Fig. S19 CLSM images of cell stained with Hoechst 33342 with irradiation. S7

Fig. S20 CLSM images of cells after long time light irradiation. S8

Fig. S21 Live/Dead staining of 4T1 cells. S8

Fig. S22 LDH release of 4T1 cells after various treatment. S9

Fig. S23 Characterization of TBMPEI dots. S9

Fig. S24 *Ex vivo* fluorescence image of mice after 24 h injection. S10

Fig. S25 Photos of tumors after 14-days treatment. S10

Fig. S26 Body-weight change of mice. S10

Fig. S27 H&E staining of mice major organs in different groups. S10

Fig. S28 Hemolytic assay of TBMPEI dots. S10

Fig. S29 Quantum yield and lifetime of TBMPE and TBMPEI S10

Supporting Figures

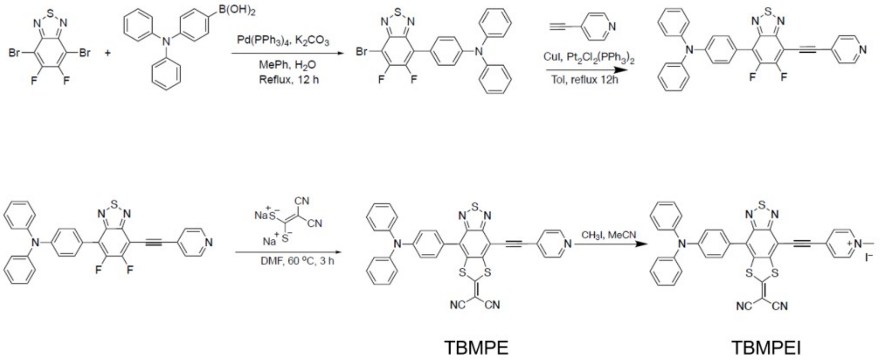

Scheme S1. Synthesis routine of TBMPE and TBMPEI.

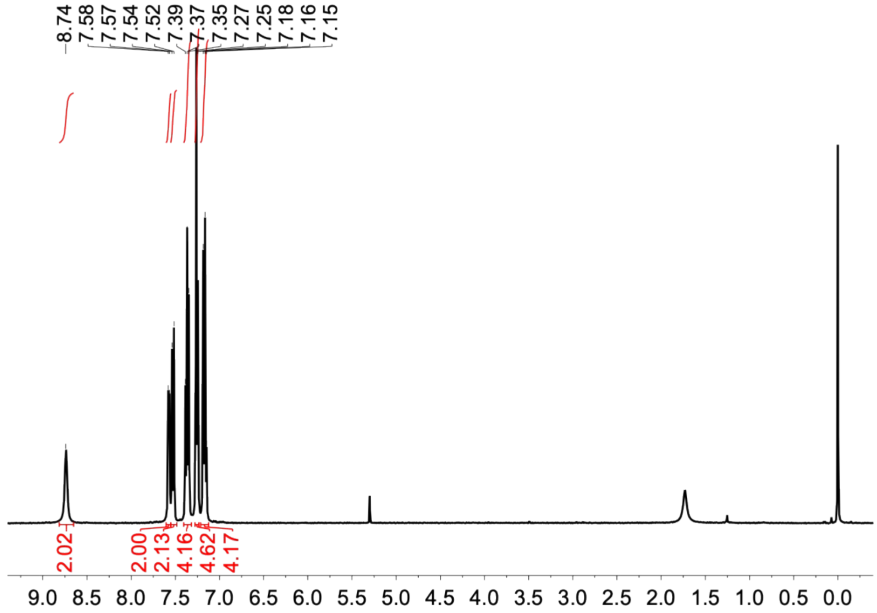

Fig. S1 ^{1}H NMR spectrum of TBMPE in $CDCl_3$.

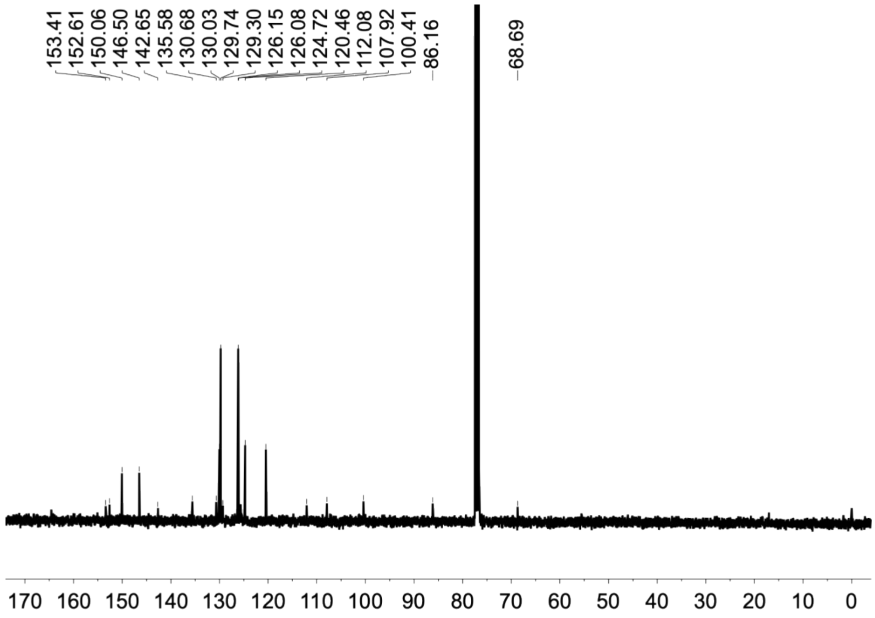

Fig. S2 ^{13}C NMR spectrum of TBMPE in $CDCl_3$.

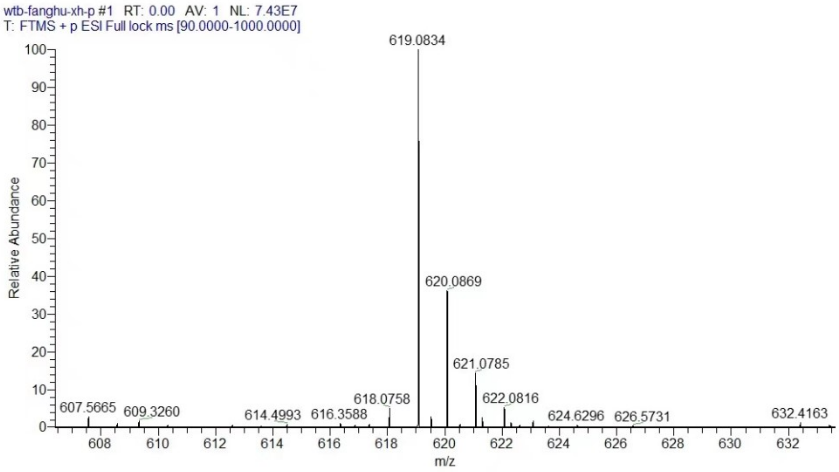

Fig. S3 HRMS spectrum of TBMPE.

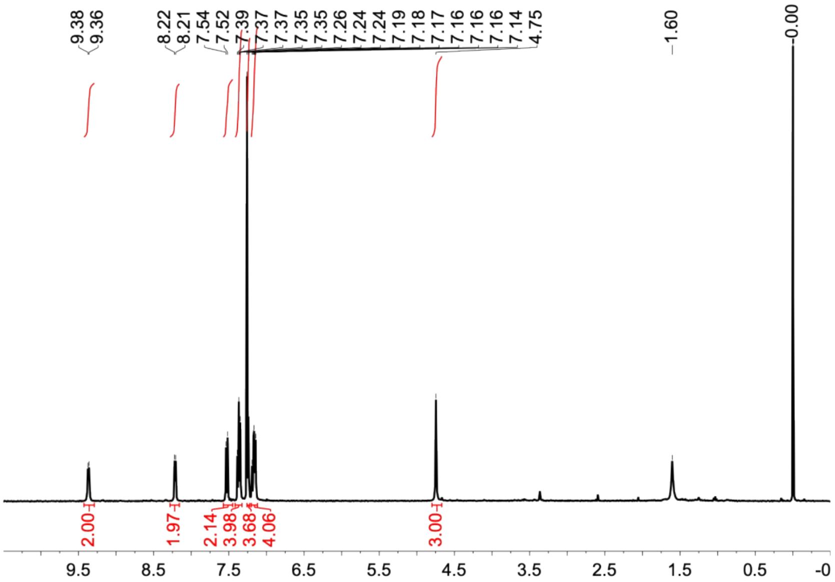

Fig. S4 ^{1}H NMR spectrum of TBMPEI in $CDCl_3$.

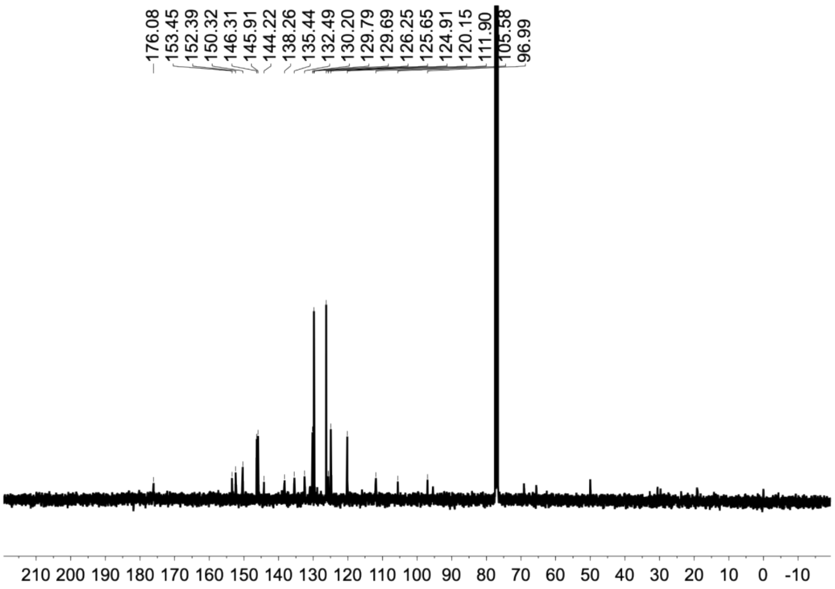

Fig. S5 ^{13}C NMR spectrum of TBMPEI in $CDCl_3$.

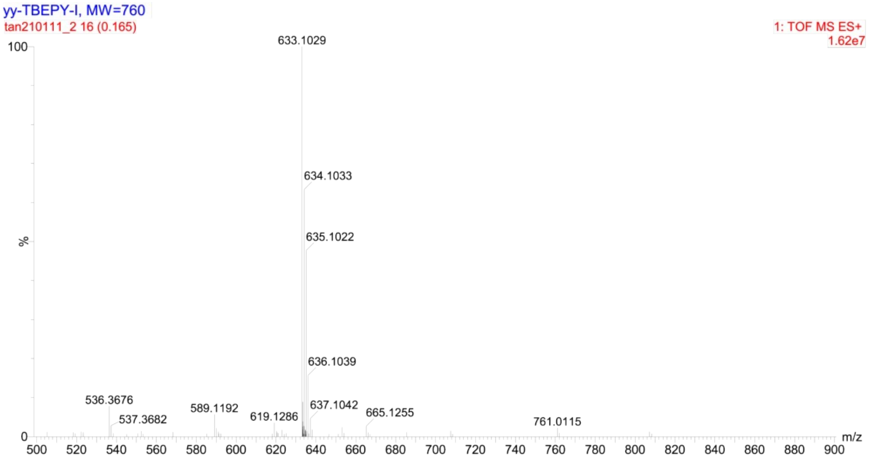

Fig. S6 HRMS spectrum of TBMPEI.

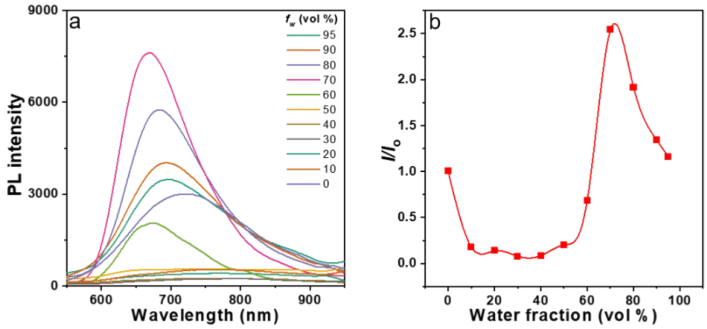

Fig. S7 (A) Fluorescence spectrum of TBMPE (10 μM) in THF/H_2O mixture with different water fraction. (F) Plots of relative PL intensity (I/I_0) of TBMPE versus water fraction.

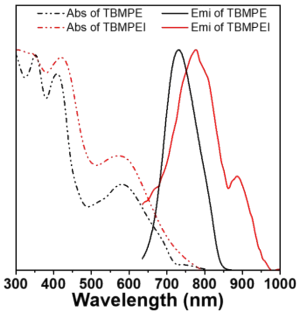

Fig. S8 Absorption and emission spectrum of TBMPE and TBMPEI in solid states.

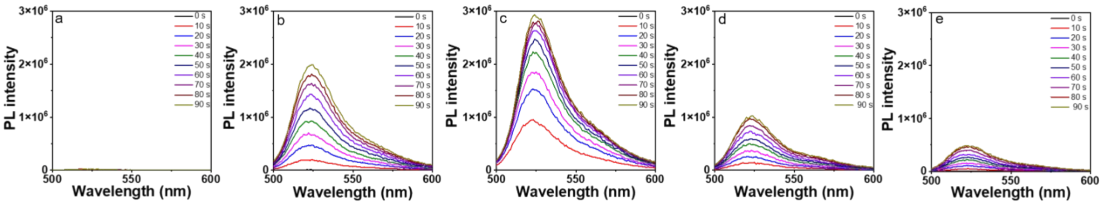

Fig. S9 Fluorescence spectrum of (a) DCFH (10 μM) and in the presence of (b) TBMPE (1 μM), (c) TBMPEI (1 μM), (d) Rose bengal (1 μM), (e) Ce 6 (1 μM) in PBS after irradiation for different time. Ex: 490 nm.

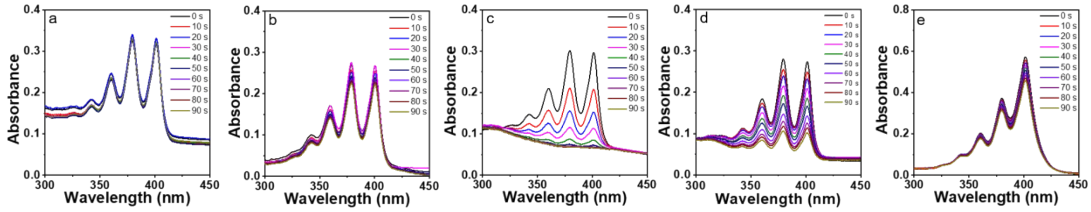

Fig. S10 Absorption spectrum of (a) ABDA (20 μM) and in the presence of (b) TBMPE (2 μM), (c) TBMPEI (2 μM), (d) Rose Bengal (2 μM), (e) Ce 6 (2 μM) in PBS after irradiation for different time.

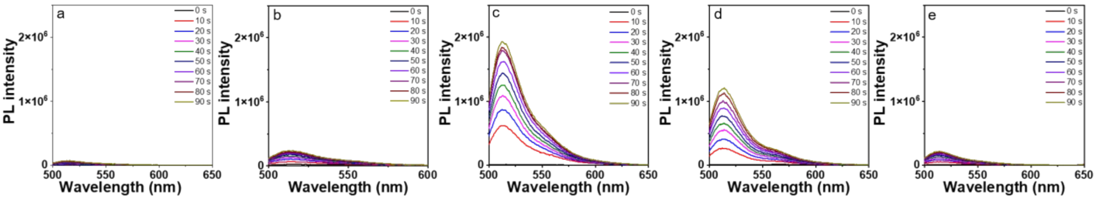

Fig. S11 Fluorescence spectrum of (a) HPF (10 μM) and in the presence of (b) TBMPE (2 μM), (c) TBMPEI (2 μM), (d) Rose bengal (2 μM), (e) Ce 6 (2 μM) in PBS after irradiation for different time. Ex:500 nm.

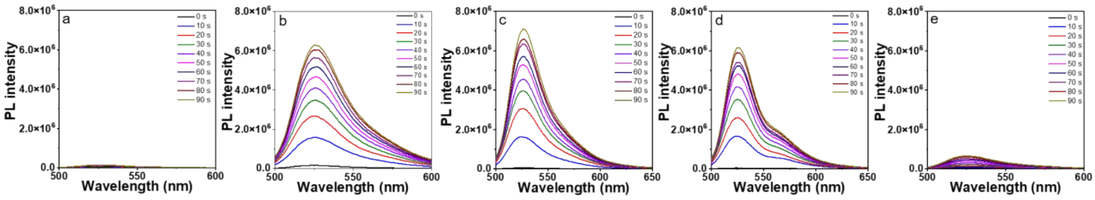

Fig. S12 Fluorescence spectrum of (a) DHR 123 (10 μM) and in the presence of (b) TBMPE (2 μM), (c) TBMPEI (2 μM), (d) Rose Bengal (2 μM), (e) Ce 6 (2 μM) in PBS after irradiation for different time. Exi: 490 nm.

TBMEP					TBMEPI				
	eV		eV	Δ E_{ST}		eV		eV	Δ E_{ST}
S5	4.0325	T5	3.1608	0.8717	S5	2.8946	T5	2.8464	0.0482
S4	3.7934	T4	2.9876	0.8058	S4	2.8435	T4	2.3960	0.4475
S3	3.5085	T3	2.5886	0.9199	S3	2.6593	T3	2.2664	0.3929
S2	3.2379	T2	2.4405	0.7974	S2	2.2088	T2	1.9398	0.269
S1	2.3425	T1	1.1389	1.2036	S1	0.9496	T1	0.9101	0.0395

Fig. S13 DFT calculations of molecular energy at various states and responding ΔE_{st}. Calculation was performed by DFT theory calculations at the m062x/6-31g* level using the Gaussian 09 program.

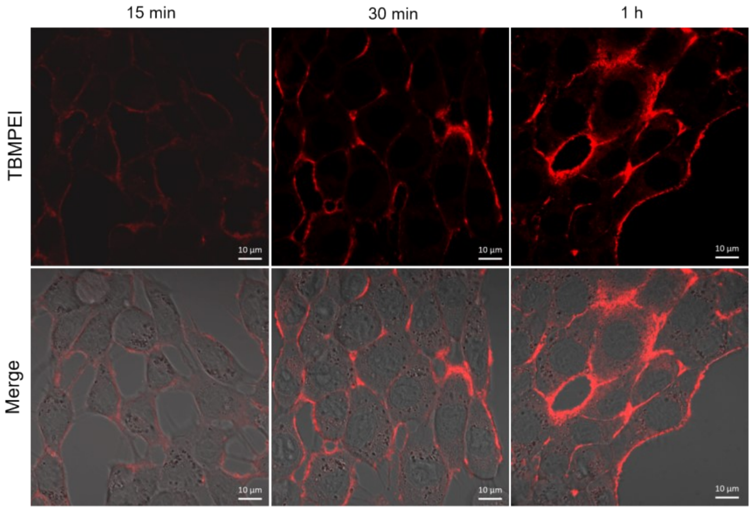

Fig. S14 CLSM images of 4T1 cells after incubation with TBMPEI for various time periods. Scale bar: 10 μm.

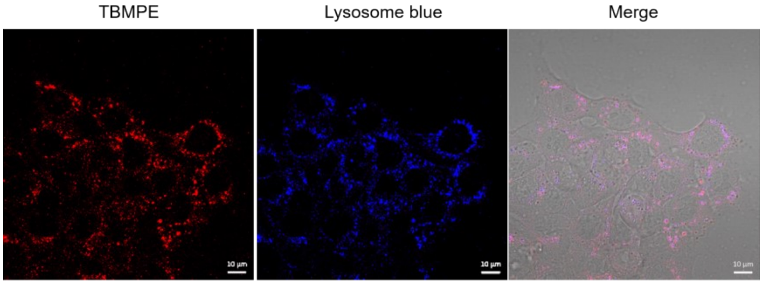

Fig. S15 CLSM images of 4T1 cells after incubation with TBMPE (10 μM, 30 min) and then co-stained with lysosome blue (2 μM, 30 min). Scale bar: 10 μm.

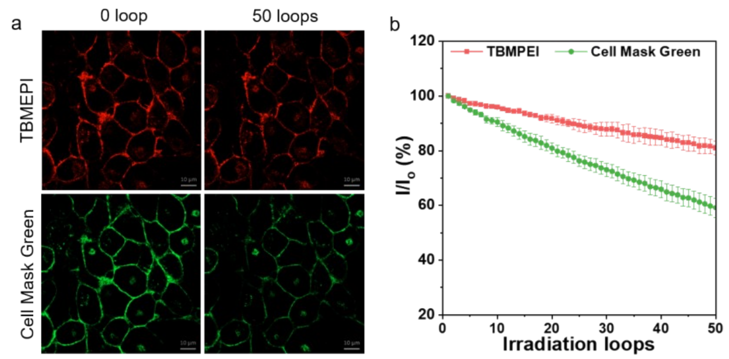

Fig. S16 Photostability comparison of TBMPEI (5 μM) and Cell Mask Green (2 μM). (a) CLSM images of 4T1 cells stained with TBMPEI and Cell Mask Green before and after 50 loops of 488 nm laser (1% intensity) irradiation.

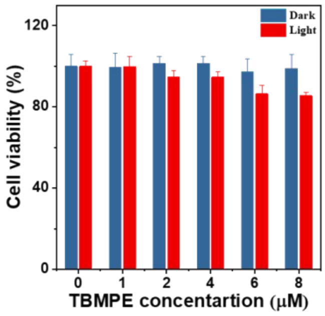

Fig. S17 Cytotoxicity of TBMPE towards 4T1 cells.

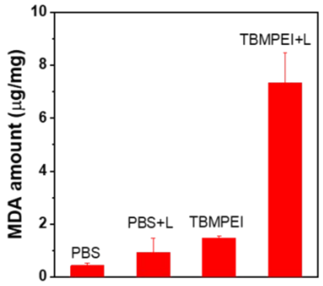

Fig. S18 MDA contents of 4T1 cells after different treatment.

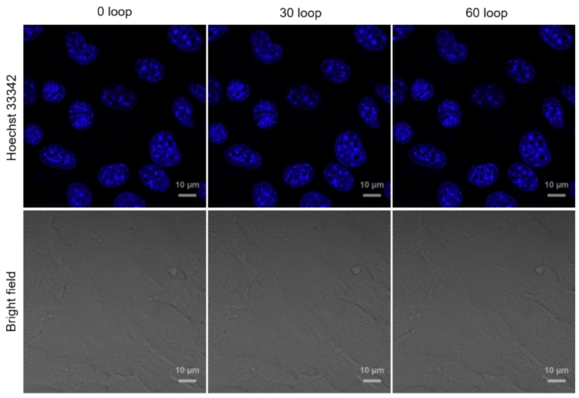

Fig. S19 CLSM images of 4T1 cells stained with Hoechst 33342 (2 μM) and under continuous 488 nm laser (3% intensity) irradiation for different loops. Scale bar: 10 μm.

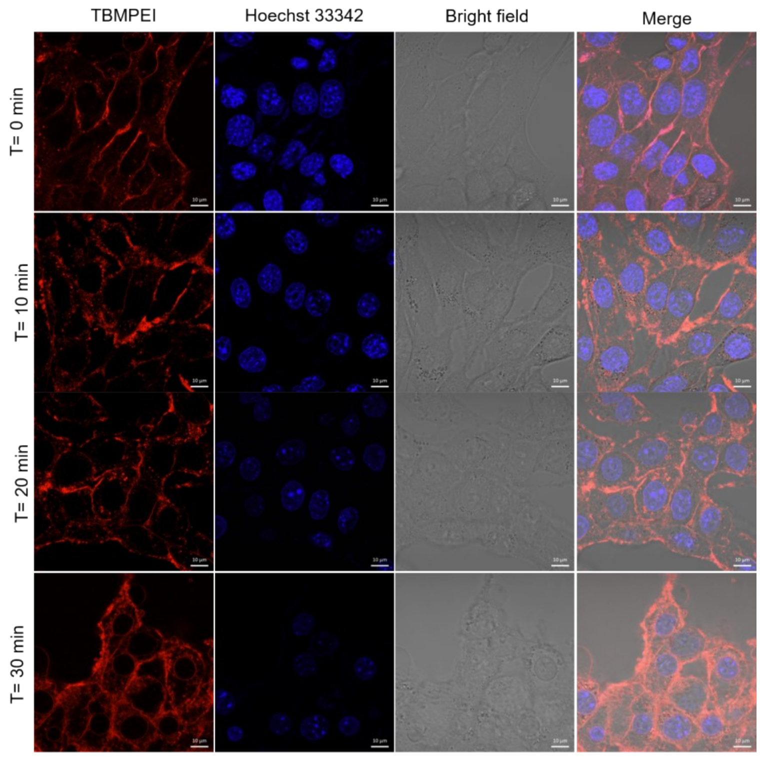

Fig. S20 CLSM images of 4T1 stained with TBMPEI (10 μM) and Hoechst 33342 (2 μM) and then irradiated with white LED lamp for various time periods. Scale bar: 10 μm.

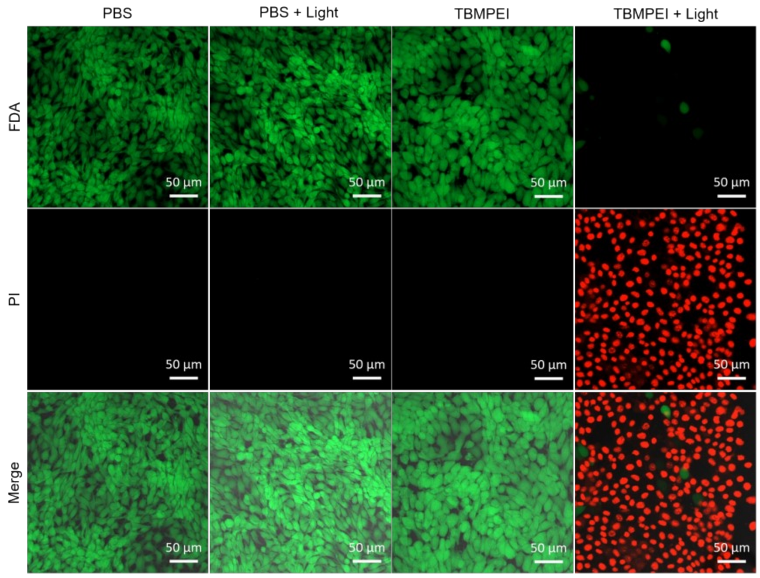

Fig. S21 Live/dead staining of 4T1 cells by FDA (6 μM, green fluorescence) and PI (10 μM, red fluorescence) after various treatments. Scale bar: 50 μm.

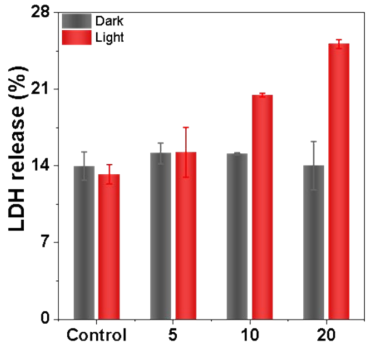

Fig. S22 LDH release of 4T1 cells after treated with various amounts of TBMPEI, 4T1 cells were chosen as the control.

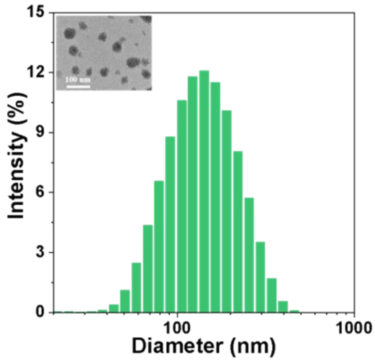

Fig. S23 Particle size distributions of TBMPEI dots in aqueous solution. Inset: TEM image of TBMPEI dots. Scale bar: 100 nm.

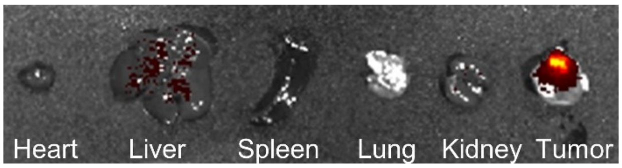

Fig. S24 *Ex vivo* fluorescence images of tumors and major organs after injection of TBMPEI dots for 24 h.

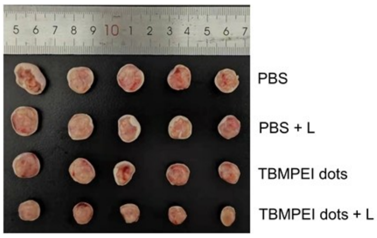

Fig. S25 Photos of tumor in various group after 14-days treatment.

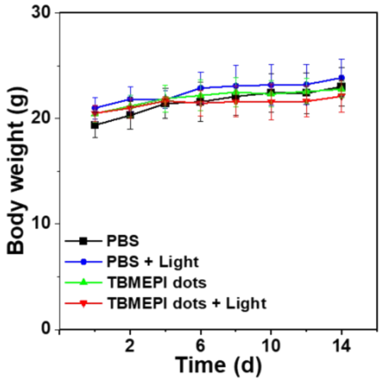

Fig. S26 Body weights change of mice in different groups during 14-day treatments.

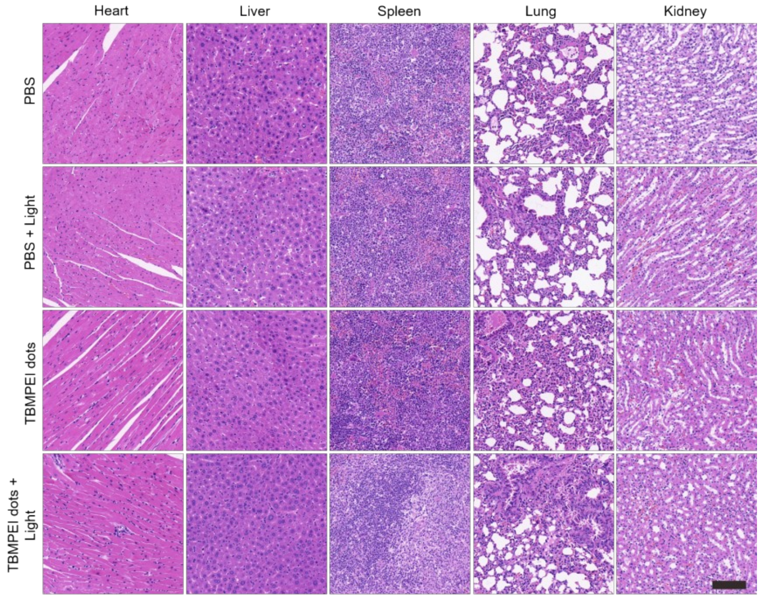

Fig. S27 H&E-stained images of tissue sections (kidney, lung, spleen, liver, and heart) of mice treated with PBS, PBS + light, TBMPEI dots, and TBMPEI dots + light. Scale bar: 200 μm.

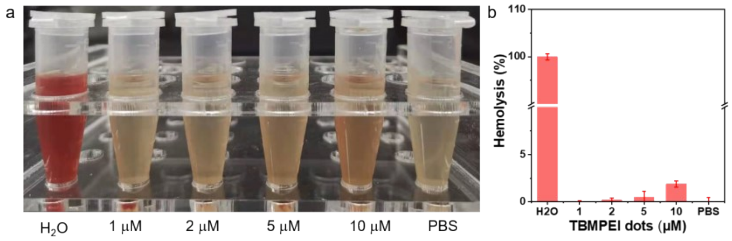

Fig. S28 Hemolysis assay of TBMPEI dots. (A) Photographs of Hemolysis assay using various materials and (B) corresponding hemolysis rates.

AIEgens	ϕ_F		α_{AIE} ($I_{aggr,max}/I_{solu}$)	τ [ns]
	Soln (ϕ_F)	Aggr (ϕ_F)		
TBMPE	1.8%	3.6%	2	1.11
TBMPEI	0.4%	0.6%	1.5	2.31

Fig. S29 Fluorescence quantum yield and lifetime of TBMPE and TBMPEI.
